# Addition of shock wave therapy to nail dynamization increases the chance of long-bone non-union healing

**DOI:** 10.1186/s10195-021-00620-9

**Published:** 2022-01-08

**Authors:** Josef Stolberg-Stolberg, Thomas Fuchs, Moritz F. Lodde, Steffen Roßlenbroich, Patric Garcia, Michael Raschke, Jens Everding

**Affiliations:** 1 Department of Trauma, Hand and Reconstructive Surgery, University Hospital Muenster, Albert-Schweitzer-Campus 1, Building W1, 48149 Muenster, Germany; 2 Department of Trauma and Reconstructive Surgery, Vivantes Hospital Friedrichshain, Landsberger Allee 49, 10249 Berlin, Germany

**Keywords:** Shock wave, Non-union, Nail dynamization, Long bones

## Abstract

**Background:**

Long-bone non-unions after intramedullary nailing can be treated by nail dynamization or focused high-energy extracorporal shock wave therapy (fESWT). The objective of this study was to assess the effect of the combination therapy of nail dynamization and fESWT on long-bone non-unions.

**Materials and methods:**

49 patients with long-bone non-unions (femur and tibia) after nailing were treated with nail dynamization (group D, *n* = 15), fESWT (group S, *n* = 17) or nail dynamization in addition to fESWT (group DS, *n* = 17). Patients were followed up for 6 months retrospectively. Furthermore, age, sex, Non-Union Scoring System (NUSS) score, time intervals from primary and last surgery until intervention and smoking status were analysed for their correlations to bone union.

**Results:**

Union rates were 60% for group D, 64.7% for group S and 88.2% for group DS, with a significant difference between group D and DS (*p* = 0.024). Successful treatment was correlated with high age (OR 1.131; 95% CI 1.009–1.268; *p* = 0.034), female gender (OR 0.009; 95% CI 0.000–0.89; *p* = 0.039), low NUSS score (OR 0.839; 95% CI 0.717–0.081; *p* = 0.028) and negative smoking status (OR 86.018; 95% CI 3.051–2425.038; *p* = 0.009).

**Conclusions:**

Data from the present study indicate that the combination therapy of nail dynamization and fESWT leads to a higher union rate than dynamization or fESWT alone.

**Level of evidence:**

Level 3.

## Introduction

Fracture healing is a complex regenerative process that usually results in scar-free bone union [1]. However, 1.9–10% of all fractures show impaired healing, with the lower leg being at particular risk: non-union rates of up to 50% are observed in cases of open fracture [2–4]. While a significant effort has been made to improve non-union treatment, the underlying processes are not well understood. Hence, the diamond concept provides a structural framework that emphasizes the importance of biological and mechanical factors in osseous union [5].

One of the most commonly performed procedures to mechanically accelerate fracture healing after nailing of long bones is nail dynamization. This comprises the removal of proximal or distal locking screws in statically locked intramedullary nails in order to reduce the fracture gap and increase the compression force. This technique was initially intended to improve union rates during normal fracture healing. It is an economical and technically simple procedure for the treatment of long-bone non-unions. However, clinical studies are yet to prove a beneficial effect [6]; reported union rates range between 30 and 90%, and clinical studies are difficult to compare [7–12]. Factors affecting treatment success include time since fracture and the callus-to-diaphysis ratio [13, 14]. Meta-analyses and systematic reviews are scarce [6].

For clinical use, the Moghaddam risk score has been developed as a means to estimate the risk for non-union formation [15]. Furthermore, the Non-Union Scoring System (NUSS) assigns specialized treatment strategies to non-unions according to their NUSS scores. Factors such as open fracture, clinical infection signs and smoking status have been identified as favouring non-union formation. The Ladder Strategy recommends the standard treatment for patients with scores of 0–25, a specialized treatment such as fESWT, growth factors or mesenchymal stromal cells for patients with between 26 and 50 points, bone resection and transport as well as microvascular flaps for patients with 51–75 points, and arthrodesis, mega-prosthesis or even amputation in patients with scores above 76 points [15, 16]. The aim of the present study was to investigate the effect of additional shock wave treatment on nail dynamization of long-bone non-unions.

High-energy focused extracorporal shock wave therapy (fESWT) is a non-invasive outpatient procedure to biologically stimulate fracture healing in non-unions [17]. Shock waves are single acoustic impulses with a rapid pressure increase followed by a tensile phase with a low amplitude generated by an electrohydraulic, electromagnetic or piezoelectric mechanism [18]. Cell and extracellular matrix stimulation induce the expression of angiogenetic and osteogenetic factors that are important for bone healing [19]. Exclusion of infection and achievement of the correct bone alignment, length, rotation and non-union gap (< 5 mm) are reported to be required for successful fESWT application. Clinical studies show union rates of between 60 and 87% and state that fESWT and revision surgery are equally effective [20–22]. However, fESWT is not widely used, and revision surgery including non-union resection, bone grafting and re-fixation remains the gold standard for most clinics and most non-unions.

Until the present work, no study had compared fESWT and nail dynamization or analysed the effect of combining fESWT and nail dynamization on long-bone non-unions. fESWT can also be applied in cases of non-union where nail dynamization is used to further stimulate bone healing. This can be done without incurring significant financial expense, increasing the surgery time or elevating the risk for complications. The aim of this study was to analyse the effect of adding fESWT to nail dynamization in diaphyseal non-union treatment, and to show its feasibility in clinical practice.

## Materials and methods

This study was conducted as a retrospective, monocentric study during 2014–2019. It was authorized by the local ethics committee (the ethics committee of the Medical Association of Westfalen-Lippe, no: 2016-160-f-S. Bone healing was defined as the presence of at least tricortical bridging callus and painless full weight bearing 6 months after trauma.

### Inclusion criteria

**Fig. 1:**
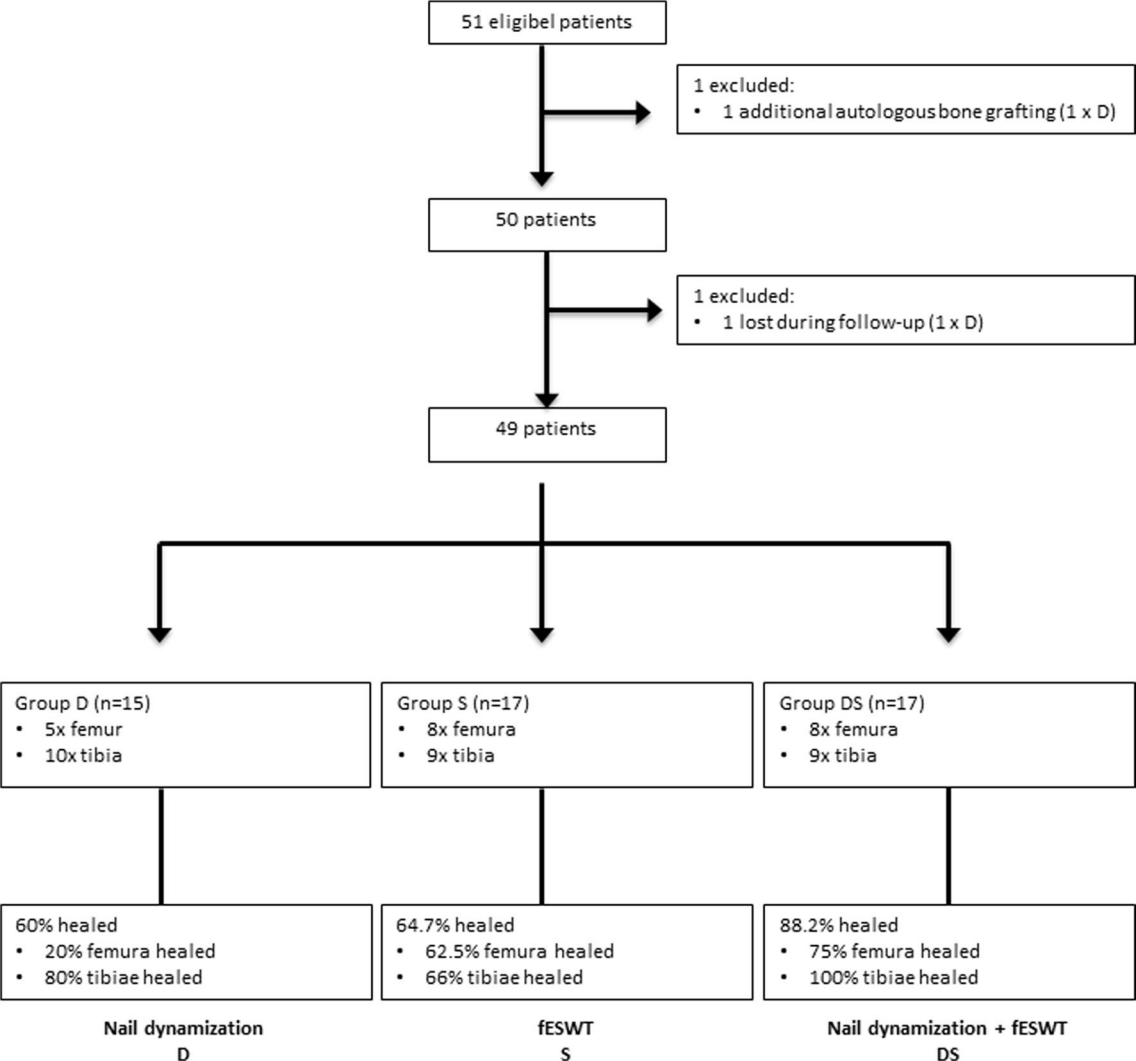
Flow diagram of the study and treatment success

The clinical information system was scanned for the search terms “shock wave” and “dynamization”. All cases were viewed manually, and only patients with long-bone non-unions (non-union gap ≤ 5 mm) after nailing of the femur or tibia who received only shock wave treatment (group S), only nail dynamization (group D) or the combination of both (group DS) were included. Patient history was scanned for signs of infection at the site of treatment and patients with elevated infectious blood parameters were excluded. Radiographic images taken at the time of treatment were examined and patients with implant loosening, axial deviation or signs of procedural implant errors were not included. All patients with additional procedures such as autologous bone grafting or bone morphogenetic protein application were excluded (Fig. 1).

Patient assignment to groups D, S, and DS was performed based on the decision of the treating senior physician. All patients agreed by written consent to the treatment procedure. Additionally, patient age, sex, AO–Mueller classification, anatomic region, size of the non-union gap, time between trauma and treatment, time since last surgery, and NUSS score were assessed at treatment (Table 1) [16].

**Table 1:** Baseline characteristics of the treated patients

	Group	Age in years	Sex	Localization	Fracture classification	Soft-tissue damage	Healed	Time between fracture and treatment in days	Time since last surgery in days	NUSS score
1	D	31	M	Femur	AO 32 C1	Closed	No	159	159	22
2	D	26	M	Femur	AO 32 B3	II° Open	No	550	357	26
3	D	26	M	Femur	AO 32 B3	Closed	No	550	550	19
4	D	54	M	Tibia	AO 42 A3	Closed	Yes	622	622	22
5	D	20	M	Tibia	AO 42 B2	III° Open	No	127	115	46
6	D	50	F	Tibia	AO 42 A1	Closed	Yes	488	488	36
7	D	53	M	Tibia	AO 42 B1	Closed	Yes	127	115	24
8	D	39	F	Femur	AO 32B1	Closed	No	235	235	18
9	D	27	M	Tibia	AO 42 C1	Closed	Yes	429	429	15
10	D	30	M	Tibia	AO 42 C2	Closed	No	156	153	11
11	D	44	M	Femur	AO 32 A3	Closed	Yes	473	473	9
12	D	58	M	Tibia	AO 42 A3	II° Open	Yes	86	86	17
13	D	21	M	Tibia	AO 42 A3	Closed	Yes	56	56	12
14	D	18	M	Tibia	AO 42 A3	Closed	Yes	137	137	10
15	D	74	M	Tibia	AO 42 C1	I° Open	Yes	138	138	16
16	S	47	F	Tibia	AO 42 A3	III° Open	Yes	293	287	30
17	S	47	F	Tibia	AO 42 A3	III° Open	Yes	293	287	30
18	S	37	M	Tibia	AO 42 C3	III° Open	No	297	302	44
19	S	52	M	Tibia	AO 42 A1	Closed	Yes	287	287	40
20	S	28	F	Femur	AO 32 A3	Closed	Yes	483	483	30
21	S	28	F	Femur	AO 32 A3	Closed	No	483	483	30
22	S	16	F	Femur	AO 32 A3	Closed	Yes	666	666	22
23	S	60	M	Tibia	AO 42 A1	Closed	Yes	383	275	30
24	S	65	M	Femur	AO 32 B3	Closed	No	169	160	32
25	S	23	M	Tibia	AO 42 B3	II° Open	No	195	191	54
26	S	50	M	Tibia	AO 42 C1	Closed	Yes	142	142	34
27	S	55	F	Femur	AO 32 A3	Closed	Yes	569	326	36
28	S	45	M	Tibia	AO 42 B3	Closed	No	821	623	36
29	S	57	M	Tibia	AO 42 C2	II° Open	Yes	376	365	38
30	S	52	M	Femur	AO 32 A3	II° Open	No	557	496	28
31	S	26	M	Femur	AO 32 B3	Closed	Yes	572	370	38
32	S	42	F	Femur	AO 32 B3	Closed	Yes	341	224	28
33	DS	39	F	Femur	AO 32 A3	Closed	Yes	128	128	22
34	DS	57	M	Tibia	AO 42 B3	III° Open	Yes	232	232	44
35	DS	43	M	Femur	AO 32 A1	Closed	Yes	426	426	30
36	DS	28	M	Tibia	AO 42 A3	Closed	Yes	289	239	24
37	DS	27	F	Tibia	AO 42 A1	Closed	Yes	382	259	26
38	DS	20	F	Femur	AO 32 C3	Closed	No	358	358	28
39	DS	46	M	Tibia	AO 42 A2	Closed	Yes	173	173	52
40	DS	65	M	Femur	AO 32 B3	Closed	Yes	383	383	34
41	DS	25	M	Femur	AO 32 B3	II° Open	No	184	182	38
42	DS	50	M	Tibia	AO 42 C3	II° Open	Yes	264	264	34
43	DS	52	M	Tibia	AO 42 A2	II° Open	Yes	146	126	42
44	DS	28	M	Tibia	AO 42 C3	Closed	Yes	150	150	32
45	DS	37	M	Femur	AO 32 B3	II° Open	Yes	141	135	34
46	DS	49	M	Femur	AO 32 A3	Closed	Yes	217	26	34
47	DS	49	F	Tibia	AO 42 A3	III° Open	Yes	335	178	44
48	DS	20	M	Femur	AO 32 A3	Closed	Yes	325	325	8
49	DS	50	M	Tibia	AO 42 C2	Closed	Yes	160	160	20

### High-energy focused extracorporal shock wave treatment and dynamization

All patients were treated under either general or regional anaesthesia. An electrohydraulic shock wave device (Lithospace Ortho®, JenaMedtech) was used for all shock wave therapies. Non-union was visualized by X-ray and the level was marked on the skin. Anatomical structures such as arteries, veins and nerves were spared by the shock waves. The shock wave depth (therapeutic volume at −6 dB: 16.6 mm × 104.2 mm) was adjusted to the soft tissue thickness. In total, 3 × 1000 impulses at 23 kV were applied with an energy flux density of 0.36 mJ/mm^2^ at 4 Hz from different angles. Ultrasonic gel allowed energy transmission. Nail dynamization was done using a sterile technique and tools provided by the manufacturer. All locking screws were removed with a single skin incision, careful soft-tissue preparation and without screw breakage. Wound suture and a sterile wound dressing were applied.

**Fig. 2:**
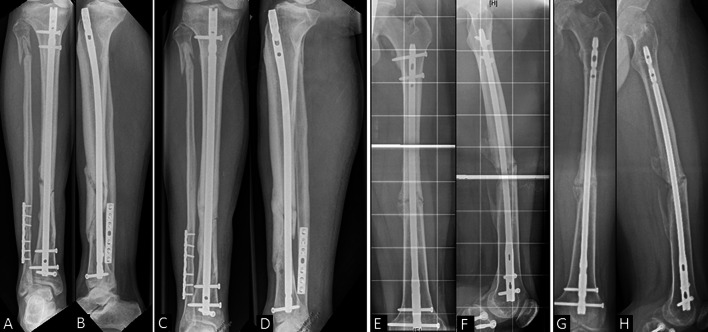
**A**–**D** Serial radiographs of a 50-year-old patient after a II° open tibia fracture showing non-union 8 months after primary surgery (**A**, **B**) and osseous union 6 months after nail dynamization and fESWT (**C**,** D**). **E**–**G** A 27-year-old patient suffered from a closed femur fracture with consecutive non-union 1.5 years later (**E**, **F**). Treatment of the clinical control with nail dynamization was not successful after 6 months (**G, H**)

Rare complications such as petechiae, neurovascular deficit or excessive pain were not found in any patient shortly after the fESWT intervention. Groups S and DS were allowed partial weight bearing for 6 weeks using crutches and thrombosis prophylaxis, while group D started full weight bearing directly after dynamization. All patients were followed up for 6 months. Non-union healing was assessed 6 months after treatment by CT scan or two plain X-rays and clinical examination by two senior orthopaedic surgeons (Fig. 2).

### Statistics

Statistical analyses were performed using IBM SPSS® Statistics 26 (IBM Corporation, Somers, NY, USA). One-way ANOVA, Kruskal–Wallis and Fischer’s exact test were performed to account for group differences. Logistic regression was conducted to analyse risk-adjusted odds ratios.

## Results

### Demographics

In total, 49 patients were followed up for 6 months during the years 2014–2019. Only long-bone shaft non-unions of the femur (AO 32) or tibia (AO 42) treated with intramedullary nailing were included in this study. Fractures and soft-tissue injuries were classified according to the AO–Mueller and Gustilo–Anderson classifications (Table 1) [23]. Altogether, group D consisted of 15 patients (5 × femur, 10 × tibia), group S consisted of 17 patients (8 × femur, 9 × tibia) and group DS consisted of 17 patients (8 × femur, 9 × tibia). The arithmetic mean ages of the 13 female and 36 male patients were 41.6 (± 13.1) and 40.6 (± 14.65) years, respectively. There was no difference in age or sex between groups (*p* > 0.05). The time interval between primary injury and non-union treatment using dynamization and/or fESWT was 289 (±202) days for group D, 407 (± 186.4) days for group S and 252.5 (± 100) days for group DS, with no significant differences between groups (Table 1). There was an average time of 275 (± 191) days, 351 (± 153) days and 220 (± 106) days for groups D, S and DS, respectively, between the last surgery and non-union treatment. Group S was treated significantly earlier than group DS (*p* = 0.045). Patients in group D had a significantly lower NUSS score of 19.5 (± 11) than those in groups S and DS: 34 (± 7.4) and 37.9 (± 10.3), respectively (*p* < 0.006). Group D had an average non-union gap of 2.2 (± 0.42) mm, that for group S was 2.9 (± 1.1) mm and that for group DS was 2.8 (± 0.72) mm, with the non-union gap in group D being significantly smaller than that in group DS (*p* = 0.029). There were no significant differences regarding smoking, soft-tissue damage or type of non-union between groups.

### Primary outcome measures

**Fig. 3:**
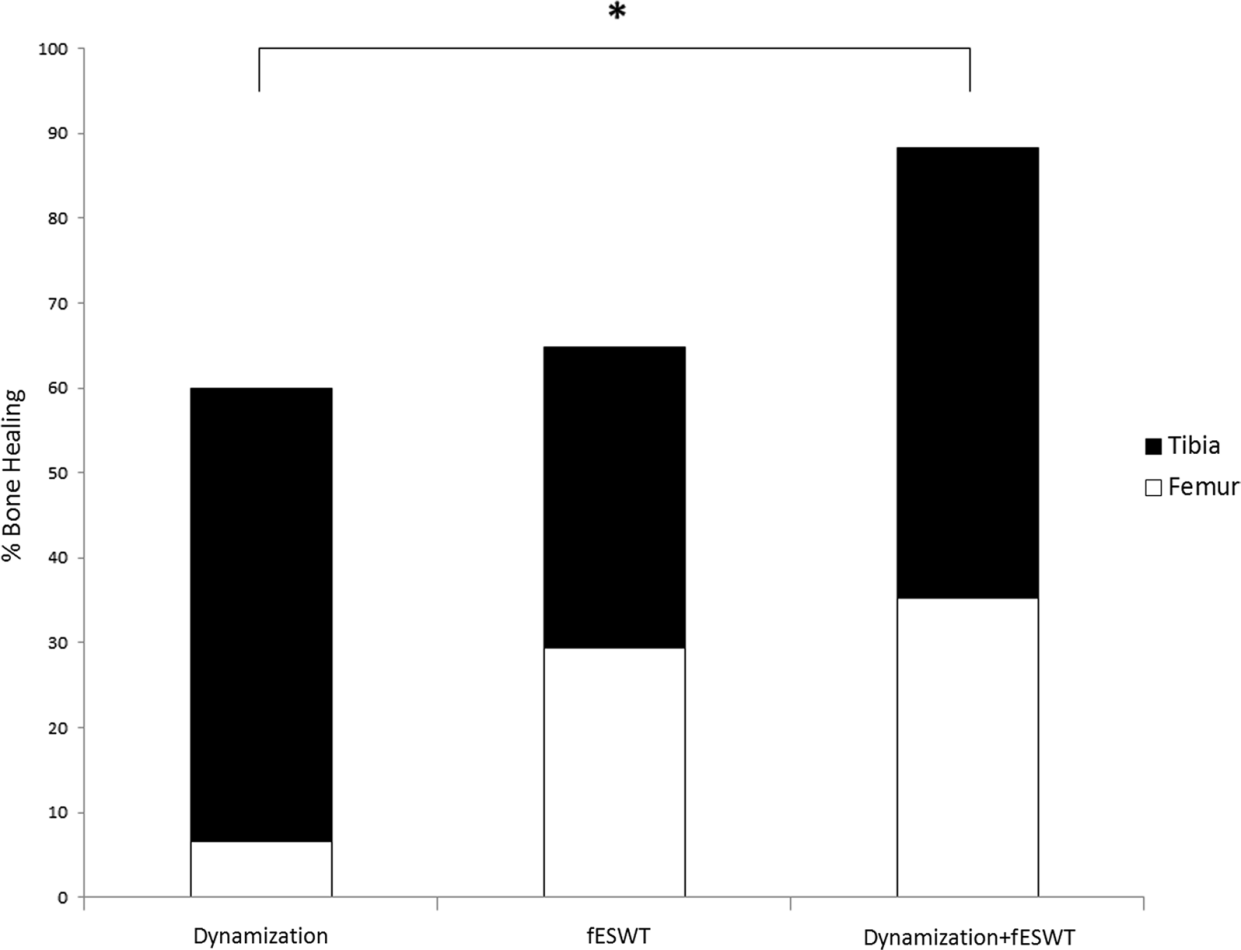
Combined nail dynamization and fESWT yielded a significantly higher union rate compared to nail dynamization alone (*p* < 0.05)

Non-union healing was assessed 6 months after intervention, and significant differences between groups were observed (*p* = 0.045). Groups D, S and DS showed union rates of 60%, 64.7% and 88.2%. There was no significant difference between groups D and S according to a risk-adjusted comparison (*p* = 0.65). However, the union rate of group DS was significantly higher than that of group D (*p* = 0.024) (Fig. 3).

### Secondary outcome measures

**Fig. 4:**
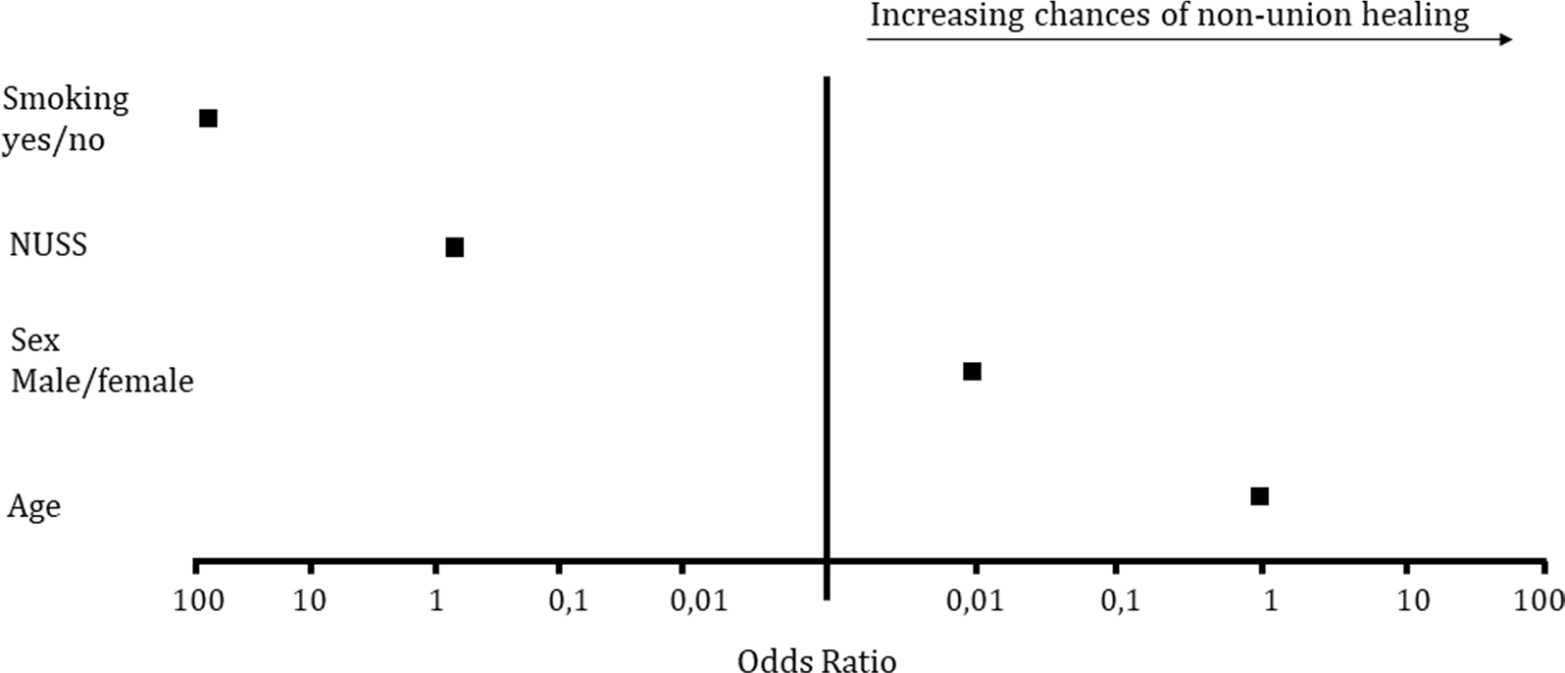
Odds ratios for bone union according to smoking status (yes/no), NUSS score, sex (male/female) and age

Taking all patients together, age (OR 1.131; 95% CI 1.009–1.268; *p* = 0.034) and sex (OR 0.009; 95% CI 0.000–0.89; *p* = 0.039) had significant effects on non-union healing, with older and female patients showing higher probabilities of bone union (Fig. 4). Increasing NUSS score was linked to a decreasing chance of non-union healing (OR 0.839; 95% CI 0.717–0.081; *p* = 0.028). Furthermore, smoking reduced the probability of bone union (OR 86.018; 95% CI 3.051–2425.038; *p* = 0.009). Time interval between accident and intervention, time interval between last surgery and intervention, type of non-union (hypertrophic, atrophic) and soft-tissue damage (open, closed) had no effect on treatment success.

## Discussion

The objective of this study was to investigate whether high-energy shock wave therapy in addition to nail dynamization improves bone healing after long-bone non-union. The diamond concept, a conceptual framework for non-union treatment, suggests that four factors—ososteogenic cells, mechanical stability, an osteoconductive matrix and osteoinductive mediators—are all essential for non-union healing. However, no study has investigated the combination of fESWT (for biological stimulation) with nail dynamization (to mechanically alter stability) to improve fracture healing [5, 24].

Originally, nail dynamization of long bones was performed 10–16 weeks after nail implantation to promote further callus modelling at the fracture site [25–27]. Recent literature, however, does not consider it mandatory, and even emphasizes harmful side effects such as limb shortening and prolonged treatment duration [10, 11, 28]. Furthermore, in vivo data on sheep and dogs with comminuted tibia fractures or unstable osteotomies did not demonstrate a beneficial effect of dynamization during normal fracture healing [29–31]. Subsequently, nail dynamization has become a specific procedure for use in cases of non-union, although the literature shows great variation in union rates [6, 32]. Vaughn et al. analysed 35 patients with dynamization of femoral and tibial non-unions. A 54% healing rate and a correlation between callus diameter at the time of dynamization and union rate was found [14]. Litrenta et al. reported an 83% tibial union rate by nail dynamization after a median of 156 days, and emphasized that a fracture gap of > 5 mm was a statistically negative factor for bone union [33]. The results of the present study show a union rate of 60% after dynamization only. In contrast to Vincenti et al., we did not observe a correlation between time from fracture until dynamization and union rate [13]. This emphasizes the need for combination treatment modalities such as nail dynamization and biophysical stimulation to further increase rates of non-union healing.

fESWT is a minimally invasive treatment option for delayed unions and non-unions [34]. Constant osseous healing rates of between 60 and 80% are found in the literature [18, 35, 36]. The 65% union rate observed in the present study after 6 months of follow-up is lower than that reported in an earlier publication from our group [18]. This might be explained by the exclusive selection of patients with intramedullary nail stabilization at the femur and tibia only in the present work, as tibia and femur fractures have been reported to have impaired outcomes compared to other bones. This is consistent with findings by Stojadinovic et al., who analysed 349 cases of delayed fracture healing and non-unions to develop a prognostic naïve Bayesian classifier model and identified the femur as well as intramedullary stabilization as negative healing predictors [37]. Furthermore, most previous studies did not analyse the NUSS score, which might also explain differences in union rate due to the nature of the specific non-union.

Regarding non-union treatment, only two studies have analyzed the effect of the combined application of treatment modalities. Cebriàn et al. included 57 cases of tibial non-union that were all treated surgically. In 22 of the cases, pulsed electromagmetic field (PMEF) therapy was also applied without achieving significantly increased healing rates [38]. Zhai et al. reported that significantly higher union rates of long-bone non-unions were obtained when applying autologous mesenchymal stem cells additionally to fESWT [39]. To the best knowledge of the authors, the present study describes the combination of fESWT, a biophysical procedure, with nail dynamization for the first time. Similar to PMEF, fESWT is a fast, inexpensive and low-risk procedure that can be easily performed during nail dynamization. The present study shows that bone union increased from 60% with dynamization alone to 88% with dynamization + fESWT. This result is made even more important by the fact that the DS group showed a significantly higher NUSS score than the D group. This shows that the combination of dynamization and fESWT significantly improves bone healing, even for more severe non-unions. The increased union rate after combined dynamization and fESWT could help to reduce treatment costs because of the reduced need for subsequent treatments and earlier social and occupational re-integration. This should more than compensate for the additional costs of fESWT during nail dynamization.

Female gender was correlated with an increased probability of bone union. Although there is no evidence in the literature that sex affects non-union healing, male patients tend to undertake higher-risk activities that can lead to high-energy fractures [40, 41]. This is consistent with the present study, in which 34% of the fractures in male patients and 23% of those in female patients were open. Interestingly, older patients had a higher probability of non-union healing. While the influence of age on bone healing is a controversial subject in the literature, most authors state that age may be a surrogate for the prevalence of other risk factors, such as fracture location, soft-tissue damage, secondary diseases such as diabetes, and the use of NSAIDs [40, 41]. The NUSS score provides a tool for comparing a heterogeneous group of patients with osseous non-unions [16, 42]. It is recommended that patients with a high NUSS score (26–50 points) should be considered for more specialized treatment, including biological stimulation with fESWT or PMEF. With their average NUSS score of 29.2 (± 10.9), the patients in the present study meet the recommended inclusion criteria [43]. Additionally, patients with higher NUSS scores showed a decreased chance of bone union, which is consistent with the literature. As expected, smoking reduced the chance of non-union healing [44].

The follow-up treatment after fESWT is a controversial topic in the literature [45]. Most authors, such as those of the current study, recommend reduced weight bearing after fESWT in order to promote local biological processes such as angiogenesis and the release of inflammatory cytokines [46–48]. All patients in groups S and DS in the present study were therefore advised to adopt partial (20 kg) weight bearing on crutches for 6 weeks. Patients in group D were allowed full weight bearing in accordance with current recommendations for early or late dynamization of femoral or tibial shaft fractures. Axial compression due to full weight bearing after dynamization has been recognized to improve fracture healing [49, 50]. According to those studies, we would expect improved fracture healing in the D group (full weight bearing) compared to the S and DS groups (20 kg weight bearing). Therefore, we have to conclude that either (i) current concepts of full weight bearing after dynamization are not accurate or (ii) the additional fESWT more than compensated for the effects of partial weight bearing, leading to a significantly higher union rate in the DS group. Further studies are needed to analyze the influence of weight bearing on a fracture after dynamization alone.

This retrospective study provides the first evidence of treatment success using the combination therapy of nail dynamization and fESWT. However, the study is limited by the number of patients (49), its non-randomized patient selection and its non-blinded and retrospective design. The NUSS score of group D was significantly lower than those of groups S and DS. Similarly, group D had a significantly smaller non-union gap compared to group DS. However, despite the lower NUSS score and smaller non-union gap in group D, the combination of nail dynamization and fESWT (group DS) resulted in a significantly higher union rate. Wide confidence intervals regarding the smoking status were observed, but there was still a statistically significant difference, with poorer outcomes for smokers. Strengths of the present study comprise the homogeneous cohort of patients with diaphyseal fractures of the femur and tibia only, the exclusion of infected non-unions, and correct implant positioning. All patients received the same primary operative treatment with intramedullary nailing.

In conclusion, the present study shows that implementation of fESWT additionally to nail dynamization significantly improves fracture healing in long-bone diaphyseal non-unions. The procedure is minimally invasive and can be performed as an outpatient procedure with excellent union rates if indicated and performed correctly. In comparison to the current gold standard of non-union treatment, which includes non-union resection, bone grafting and re-osteosynthesis, we present a cost-effective, low-risk alternative treatment option. However, to confirm our results, future randomized prospective and blinded studies are necessary to create a higher degree of clinical evidence.

## Data Availability

The datasets used and analysed during the current study are available from the corresponding author on reasonable request.

## Abbreviations

fESWT: Focused high-energy extracorporal shock wave therapy
NUSS: Non-Union Scoring System
Group D: Group treated with nail dynamization
Group S: Group treated with fESWT
Group DS: Group treated with nail dynamization in addition to fESWT
PMEF: Pulsed electromagmetic fields
